# The complete mitochondrial genome of the lizard subspecies, *Phrynocephalus vlangalii vlangalii* (Reptilia, Squamata, Agamidae)

**DOI:** 10.1080/23802359.2017.1419087

**Published:** 2018-02-10

**Authors:** Kun Yang, Yubin Wo, Yulong Zhou, Yuanting Jin

**Affiliations:** Zhejiang Provincial Key Laboratory of Biometrology and Inspection & Quarantine, College of Life Sciences, China Jiliang University, Hangzhou, China

**Keywords:** Lizard, mitogenome, *Phrynocephalus*

## Abstract

The complete mitochondrial genome was sequenced from the toad-headed lizard, *Phrynocephalus vlangalii vlangalii.* The overall length of mitogenome is 16,319 bp, including 22 tRNA, 13 protein coding genes, 2 rRNA genes, and 2 control regions. The gene order and content were same with the published congeneric mitogenomes, besides the small portion between *tRNA-Pro* and *tRNA-Phe*.

We report the complete mitogenome of *Phrynocephalus vlangalii* from Geermu, Western Qinghai-Xizang (Tibetan) Plateau with GenBank Acc. MF039058. The specimen was stored in College of Life Sciences, China Jiliang University. The PCR and sequencing method followed the description (Liao and Jin 2016). The great mass of the genes are coded on the H-strand, and some of others are encoded on the L-strand, containing tRNA-Gln (CAA), tRNA-Ala (GCA), tRNA-Asn (AAC), tRNA-Cys (UGC), tRNA-Tyr (UAC), tRNA-Ser (UCA), tRNA-Glu (GAA), tRNA-Pro (CCA), and ND6. ND2 starts at ATT; COI, COII, ATP8, ATP6, COIII, ND4L, ND4, ND5, and Cytb regard ATG as start codon; ND6 starts at GTG; ND1 starts at ATA. ND2, ATP8, ND4L, ND4, and ND5 stops at TAA; ND1 and COI stop at AGA; ND6 stops at AGG. Other protein-coding genes (COII, ATP6, COIII, ND3, and Cytb) stop at a single stop nucleotide T.

Compared with other viviparous *Phrynocephalus* (Chen et al. 2014; Fu et al. 2016; Liao and Jin 2016; Tong and Jin 2016; Zhu et al. 2016), the segment that contains two control regions (*CR*, *tRNA-Pro, tRNA-Phe, CR*) of *P. v. vlangalii* has some difference with *P. theobaldi orientali* (*CR, tRNA-Phe, tRNA-Pro, CR.* GenBank Acc. KJ551842).

Bayesian analyses were performed in BEAST (v1.8.2) to infer the mitogenomic tree using *P. przewalskii* as outgroup. The above-published mitogenomes of *Phrynocephaus* viviparity including the well aligned fragments with 15,743 bp in length were included into analyses. The GTR + I + G substitution model selected by Akaike information criterion (AIC) was included to account for the concatenated sequences used into analyses. The phylogenetic topology (Figure 1) was robust while identical topology was obtained by different partitioning strategies as well as by using other phylogenetic inferences (i.e. maximum likelihood tree and Neighbor-Joining Tree in MEGA 7.0). The estimated topology supported a previous published phylogenetic inference including all *Phrynocephalus* viviparity (Jin and Brown 2013).

**Figure 1.**
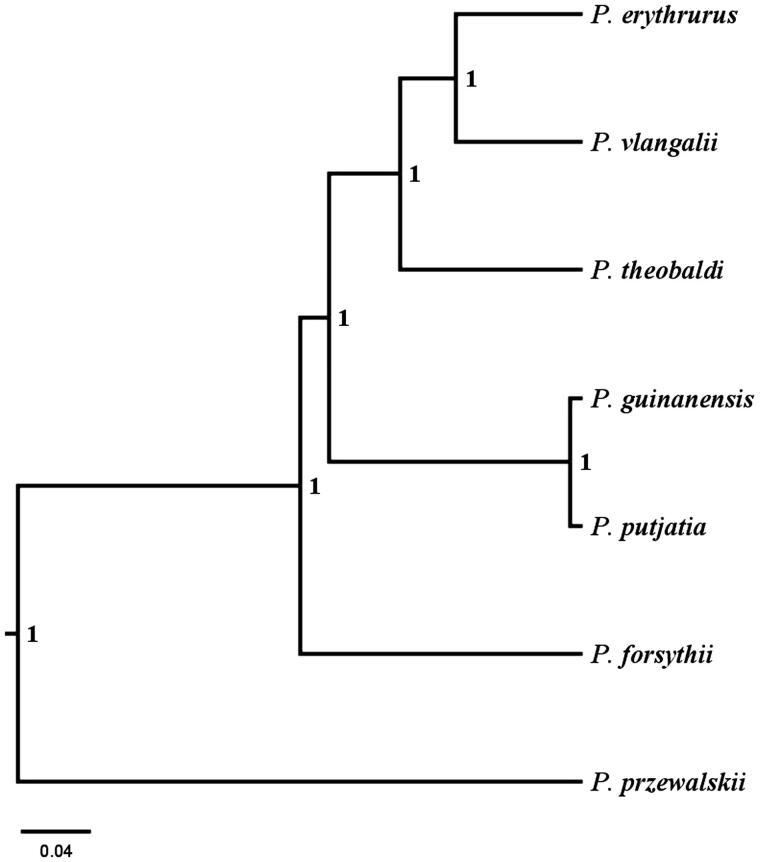
Bayesian phylogenetic inference among *Phrynocephalus* viviparity. The numbers near the nodes corresponded to the posterior probabilities.
